# De novo double-hit B-cell precursor leukemia/lymphoma - an unusual presentation as peritoneal lymphomatosis

**DOI:** 10.4322/acr.2021.278

**Published:** 2021-05-06

**Authors:** Balamurugan Thirunavukkarasu, Jayanta Samanta, Prateek Bhatia, Amanjit Bal

**Affiliations:** 1 Post Graduate Institute of Medical Education & Research, Departments of Histopathology, Chandigarh, India; 2 Post Graduate Institute of Medical Education & Research, Department of Gastroenterology, Chandigarh, India; 3 Post Graduate Institute of Medical Education & Research, Department of Pediatrics, Chandigarh, India

**Keywords:** Precursor B-Cell Lymphoblastic Lymphoma, Peritoneal neoplasms, c-myc, Ascites

## Abstract

Peritoneal lymphomatosis (PL) is a rare presentation of extranodal precursor leukemia/lymphoma. The presentation is often non-specific, leading to delayed diagnosis and treatment. In this case, though the preliminary diagnosis was established on ascitic fluid cytology, the disease progressed rapidly, leading to demise before initiating chemotherapy. Immunophenotyping and molecular studies, performed later, established a diagnosis of de novo B-cell precursor leukemia/lymphoma with *MYC*, *BCL2* rearrangements (Double-hit lymphoma). *MYC*, *BCL2* rearrangements are rarely reported in precursor B-lymphoma/leukemia which carry dismal prognosis. In this report, we illustrate autopsy findings of PL in an elderly gentleman who presented with ascites for evaluation.

## INTRODUCTION

Peritoneal lymphomatosis (PL) is a rare presentation of extranodal Non-Hodgkin lymphoma (NHL). Since the clinical presentation of PL is non-specific, the diagnosis is often delayed. Peritoneal carcinomatosis accompanied by malignant ascites is relatively common; however, ‘peritoneal lymphomatosis’ is rarely reported in the literature.^1^ Differentiation of PL from other morbid entities with similar imaging features such as tuberculous peritonitis, peritoneal carcinomatosis, and peritoneal mesothelioma is difficult without a histological diagnosis.^2^ In the present genomic era, the appropriate immunohistochemical and molecular workup of lymphoma is indispensable for guiding therapy and prognostic information. The 2016 revised World Health Organization (WHO) classification of lymphoid neoplasms has included the category of high-grade B cell lymphomas (HGBLs) with combined MYC and BCL2 and/or BCL6 rearrangements termed as double-hit (DH) or triple-hit (TH) respectively.^3^^,^^4^ DH is usually reported commonly in diffuse large B-cell lymphoma (DLBCL), less frequently in follicular lymphomas and rarely in B-lymphoblastic lymphoma/leukemia (B-LBL).^4^^,^^5^ There is limited information on cases of B-LBL with *MYC* and *BCL2* rearrangement. In addition, there are cases of HGBL which can express Tdt rendering a diagnostic dilemma. Herein, we present autopsy findings of an elderly gentleman who presented with ascites for evaluation.

## CASE REPORT

A 55-year-old gentleman presented with abdominal distension to the gastroenterology service. The distension was insidious in onset, along with decreased urine output over the last 20 days. On examination, the patient had tense ascites with bilateral pitting pedal edema. There was no history of jaundice, hematemesis, melena, altered sensorium, fever, night sweats, or weight loss. Ultrasound revealed ascites with mild hepatomegaly and hydroureteronephrosis of the right kidney. Upper gastrointestinal endoscopy did not reveal any varices. Hepatotropic viruses’ markers were negative. His renal function tests were deranged. Blood urea was 128 mg/dl (reference range [RR]; 15-40mg/dl) and serum creatinine was 2.9 mg/dl (RR; 0.6-1.2mg/dl). The liver function test on admission day was as follows: Total bilirubin: 0.4 mg/dl (RR: 0.2 - 1.2 mg/dl), conjugated bilirubin 0.2 mg/dl (RR: 0 - 0.3 mg/dl); AST: 37 (RR: 2-40 U/L) ALT: 32 (RR: 2-41 U/L) alkaline phosphatase: 62 IU/L (RR; 44-147 IU/L). However, terminally (3 days after admission), the AST/ALT/ALP were 408/90/2042 IU, respectively. Abdominal ultrasonography did not reveal features of acute Budd-Chiari syndrome such as thickened walls, intraluminal echogenicity, and compressed hepatic veins or inferior vena cava.

The ascitic fluid analysis revealed a high serum ascites albumin gradient (SAAG) ascites (1.2 g/dl). Initial ascitic tap showed sheets of polymorphs, and the subsequent cytology showed numerous intermediate size lymphoid cells with a high nuclear/cytoplasmic ratio. Immunocytochemistry showed lymphoid cells strongly positive for CD45, CD10, Tdt, and C-MYC. Focal positivity for CD20, CD19, CD79a was seen in these atypical lymphoid cells, and a working diagnosis of B-cell lymphoblastic lymphoma/leukemia was kept. Peripheral blood smear did not show blasts. CSF tap did not show atypical cell infiltration. CECT abdomen and PET-CT scan were deferred due to deranged renal function test.

Differentials for ascites were considered based on the serum-ascites-albumin gradient (SAAG). Cutoff of >1.1mg/dl is considered as high SAAG, which includes conditions like cirrhosis, heart failure, Budd-Chiari syndrome, sinusoidal obstruction syndrome. Low SAAG includes malignancy, infection, and pancreatitis, among others. The ultrasonography, endoscopy, and hepatic viral markers were negative for cirrhosis, varices, and viral hepatitis, respectively; enabling a rapid ascitic fluid malignant cytology analysis. It is interesting to note that despite being a case of PL, this case showed high SAAG ascites.

Since the initial two ascitic fluid taps showed neutrophils, the patient was empirically started on IV cefoperazone-sulbactum, oral acyclovir, oral fluconazole and twice weekly cotrimoxazole. Low molecular heparin was given as deep venous thrombosis (DVT) prophylaxis. Fluconazole and cotrimoxazole were withheld later in view of worsening liver function tests (LFT), and acyclovir was also stopped in view of renal failure. Pre-emptive steroids and chemotherapy could not be started because of the poor general status of the patient.

The patient’s renal and liver function deteriorated, and he had a sudden cardiac arrest and could not be revived despite resuscitation efforts. A complete autopsy was performed after obtaining informed written consent from the patient’s next of kin.

## AUTOPSY FINDINGS

**Figure 1:**
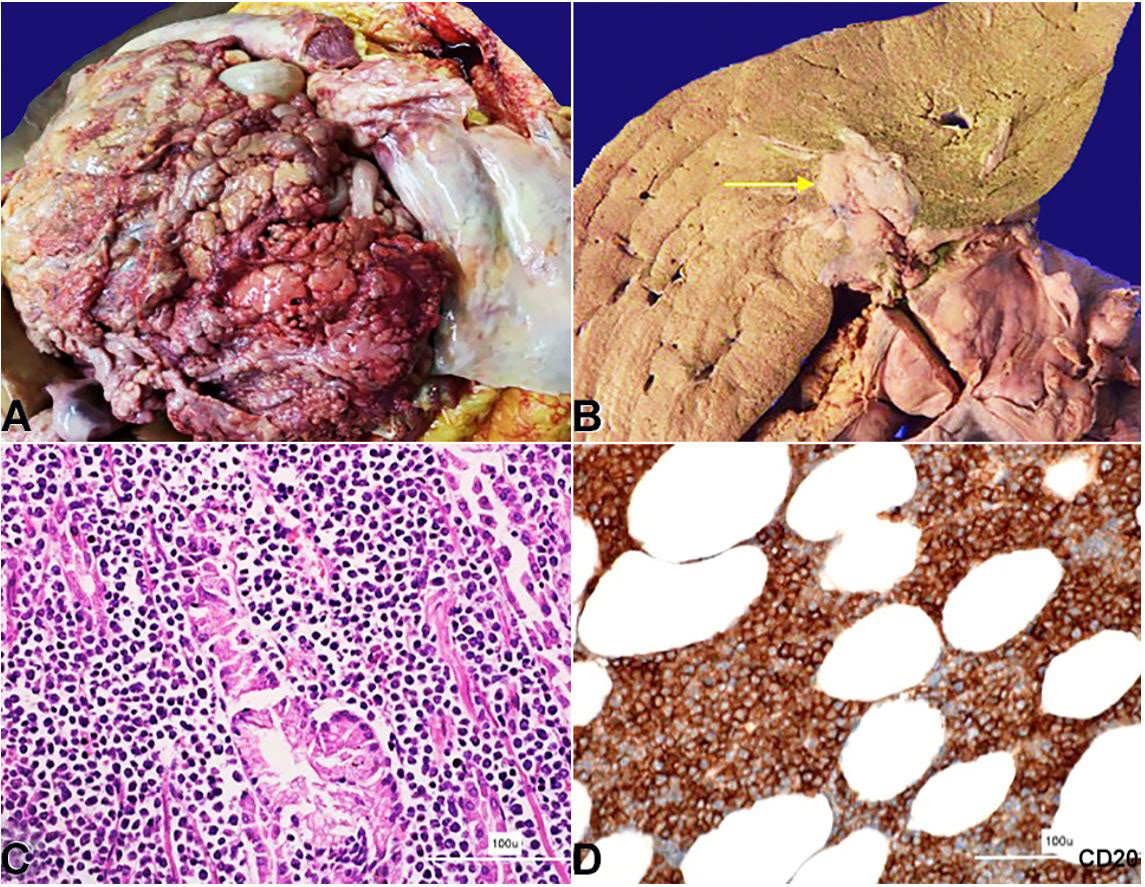
**A** – Gross view of the abdominal cavity showing diffuse nodular thickening of the peritoneum; **B** – Gross view of the liver’s cut surface showing infiltration of the tumor (yellow arrow); **C** and **D** – Photomicrographs of the tumor; **C** – Tumor cells infiltrating the gastric antral glands (H&E, 200X); **D** – Photomicrograph of the omentum showing tumor cells diffusely positive for CD20 (200X).

The peritoneal cavity yielded 2 liters of straw-colored fluid. The mesentery and the omentum appeared thick and nodular, forming adhesions to the intestinal wall (Figure 1A). Either side of the diaphragm showed similar greyish white deposits. The liver weighed 2300 g (RR;1330-2100g) and was diffusely enlarged. The porta hepatis showed a greyish white mass measuring 4x3x2cm infiltrating the hepatic parenchyma (Figure 1B). This tumor also infiltrated the pancreas, stomach, the serosal surfaces of the small and large intestine and right kidney. A similar deposit was seen in the periadrenal fat. No significant lymph node enlargement was noted. The heart and brain were grossly and microscopically unremarkable.

The microscopy revealed relatively uniform-appearing atypical lymphoid cells with blastoid morphology. These cells were infiltrating the peritoneum, porta hepatis, renal pelvis, serosa and mucosa of the gastrointestinal tract (Figure 1C). The cells were intermediate-sized with round to convoluted nuclei with condensed nuclear chromatin, indistinct nucleoli, and scanty cytoplasm. These cells were positive for CD45, CD20, CD10, TdT, CD99 (dot-like), Bcl2 (>70%), and C-MYC (>40%) while they were negative for CD3, CD5, CD34, CD23, Bcl6, Mum1, Cyclin D1 and SOX11 (Figure 1 D, 2222D).

**Figure 2:**
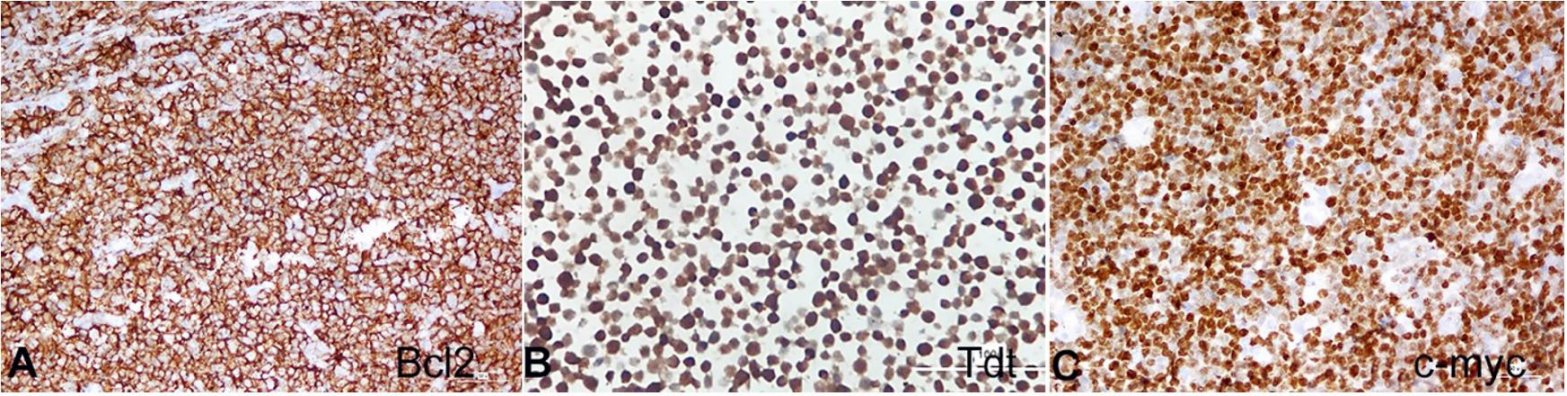
Photomicrograph of the tumor showing in **A** – Bcl2 positive (200X); **B** – Tdt positive (200X); **C** – positive c-myc (200X).

**Figure 3:**
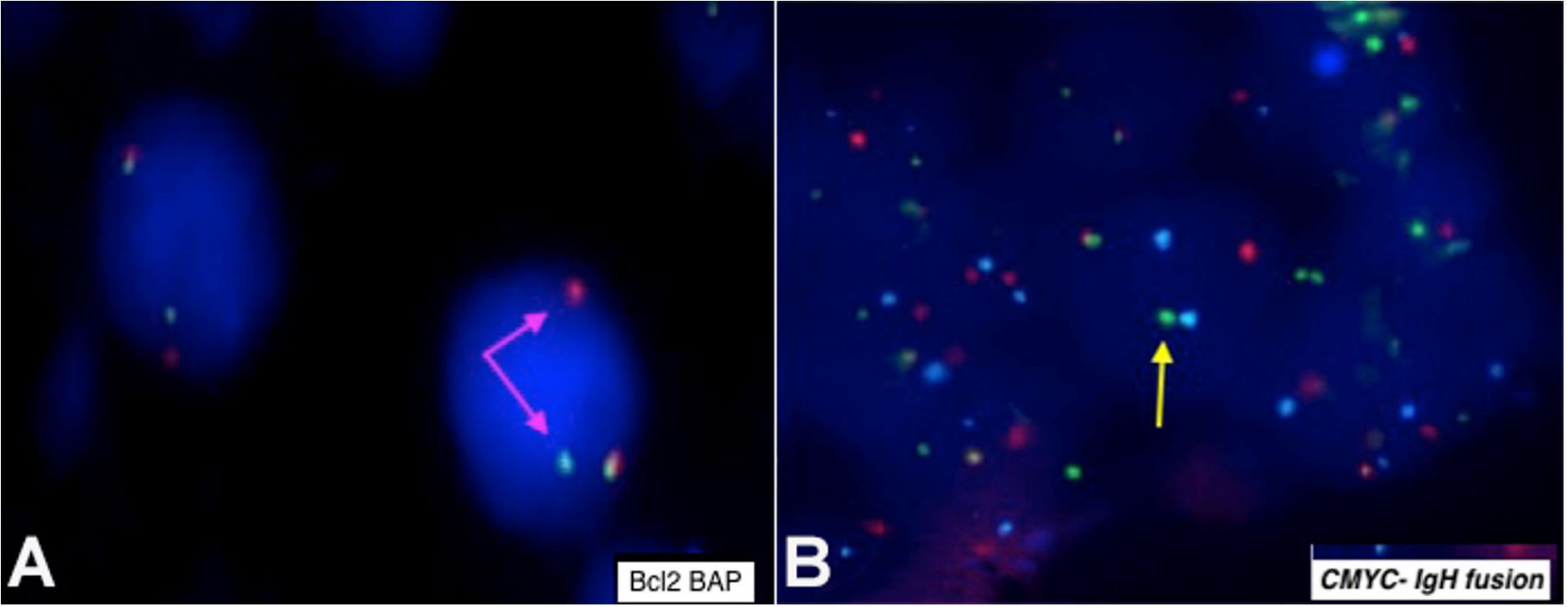
**A** – *BCL2* Dual Color break-apart rearrangement probe showing one orange, one green, and one orange/green fusion (1O1G1F) signal pattern indicating *BCL2* rearrangement (Pink arrows indicating split signals); **B** – *IGH/MYC*/CEP 8 Tri-Color Dual Fusion Probe hybridized to an abnormal nucleus showing one fusion (1F) one orange (01) one green (G1) and 2A signal pattern indicating *CMYC-IGH* fusion (yellow arrow indicating fused signal).

Immunophenotypic diagnosis of B-lymphoblastic lymphoma/leukemia was considered. The fluorescence in-situ hybridization (FISH) was positive for *BCL2* rearrangement (18q21) (Vysis LSI *BCL2* Dual Color Break Apart Rearrangement Probe) (Figure 3A) and C*MYC-IGH* fusion (IGH/MYC/CEP 8 Tri-Color Dual Fusion Probe) (Figure 3B).

**Figure 4:**
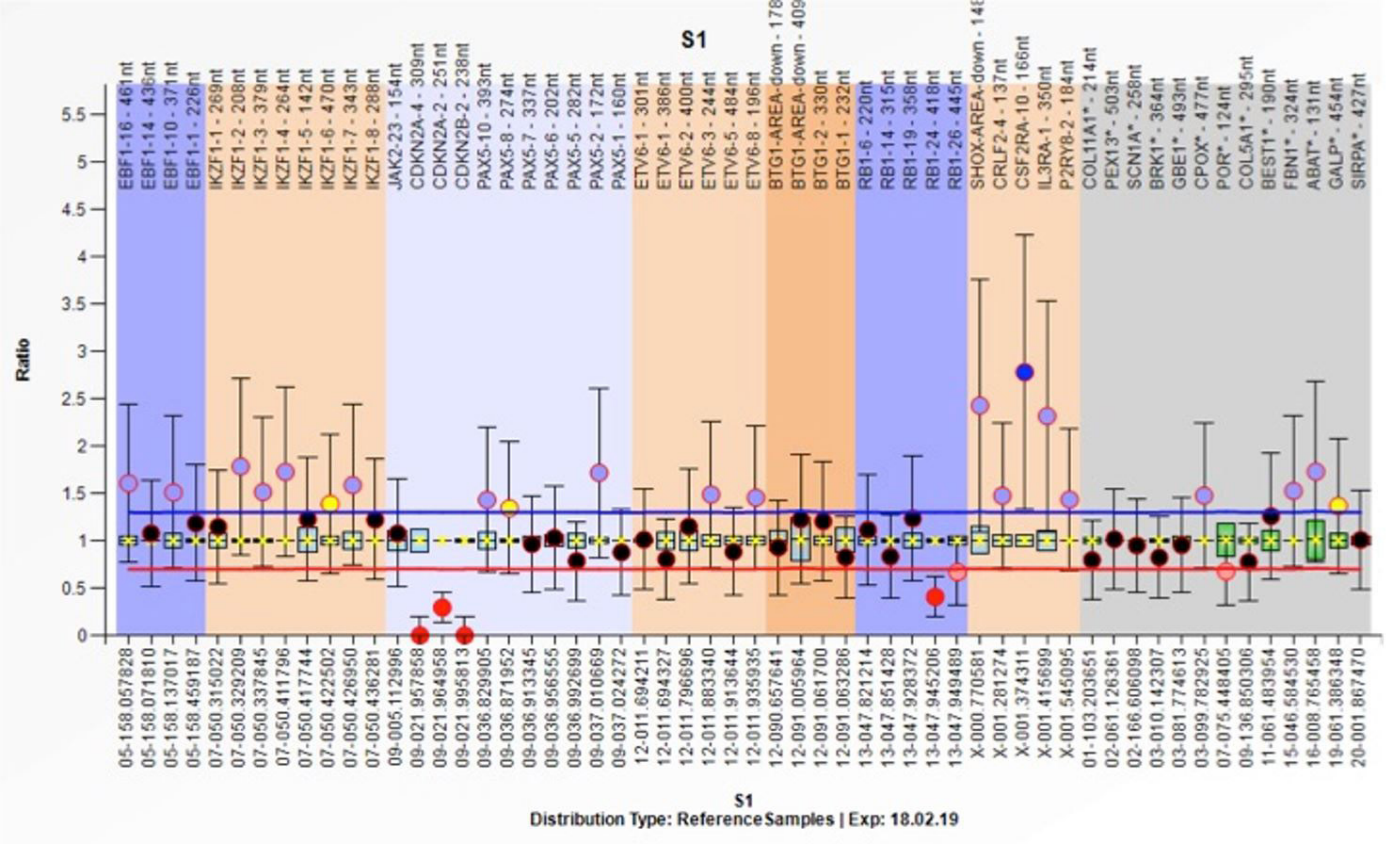
Multiplex ligation-dependent probe amplification revealed homozygous deletions of Exon 2 & 4 of *CDKN2A* and Exon 2 of *CDKNA2B*.

However, FISH was negative for *BCL6*, *BCR-ABL-1*, *KMT2A* translocation, *ETV6-RUNX1* translocation, and chromosome-21 amplification. Real-time PCR was negative for *CRLF2* overexpression. Copy number abnormalities revealed homozygous deletions of Exon 2 & 4 of *CDKN2A* and Exon 2 of *CDKNA2B* (Figure 4). Bone marrow biopsy was normocellular with adequate representation of all marrow elements. There was no infiltration by lymphoma or involvement by leukemic process. The peripancreatic and peribiliary lymph nodes did not show the lymphomatous process. The autopsy did not reveal features of hepatic venous outflow obstruction. There was no evidence of pulmonary thromboembolism. There was focal subpleural infiltration through the diaphragm (direct extension) by lymphoma on the right side.

## DISCUSSION

Non-Hodgkin lymphoma presents primarily as an extranodal disease in 25-40% of cases.^6^ It can affect organs and sites such as the biliary tree, liver, spleen, gastrointestinal tract, adrenals, peritoneal cavity, among others. Peritoneal surfaces can be involved by malignancies from all three cell-lineages: epithelial (carcinomatosis), mesenchymal (sarcomatosis), and lymphoid (lymphomatosis). Peritoneal lymphomatosis (PL) is defined as the “intraperitoneal spread of lymphoma”. It can be seen either in association with a primary visceral site of involvement, or without any visceral involvement.^7^^,^^8^ The route of dissemination is postulated to be through visceral peritoneal surfaces, gastrocolic ligament, and transverse mesocolon. PL is an exceptional clinical scenario, which is frequently associated with primary gastrointestinal NHL (high grade) and is radiologically identical to peritoneal carcinomatosis. Diffuse large B-cell lymphoma (DLBCL) is the most common lymphoma presenting as PL. Multiple patterns of PL include infiltrative mass, distinct nodular, and ascites.^8^ In the present case, there was a diffusely infiltrative mass presenting with ascites.

Based on the immunohistochemical and molecular workup, the present case was a de novo B-LBL with *MYC*, *BCL2* rearrangements. Double-hit/triple-hit lymphoma is characterized by *MYC* (8q24) rearrangement in combination with a *BCL2* (18q21) and/ or a *BCL6* (3q27) rearrangement.^9^^,^^10^ Double-hit lymphomas (DHL) are rare and represent 4-8% of all diffuse large B-cell lymphomas. They tend to occur in extranodal sites. Four morphological variants have been described in DHL; DLBCL-like, features previously designated as B-cell lymphoma unclassifiable (BCL-U) in 2008 WHO classification, Burkitt lymphoma-like and lymphoblastic lymphoma-like .^4^^,^^11^

There are few case reports of B-ALL with double hit genetics reported in literature.^12^^,^^13^ In most of those cases, the blasts were L3 type (French-American-British classification) and majority of them had acute leukemia like presentation. Few of the cases had a leukemic transformation from DLBCL. Conversely, on the other end of the spectrum, there can be acute lymphoblastic leukemia-like high grade B-cell lymphoma with *MYC* and *BCL2* and/or *BCL6* rearrangement lacking Tdt and other immaturity markers.^14^ Ok et al.^15^ described a series of 13 cases of high-grade B-cell lymphomas with TdT expression grouped into three categories. These included cases of de novo high-grade B-cell lymphoma with Tdt expression as well as blastic transformation of a low grade B-cell lymphoma which acquired Tdt during relapse. Features that favoured HGBL over LBL were positivity for BCL6 and monotypic surface immunoglobulin. All the cases showed dismal outcome, despite appropriate therapy.

In the present case, copy number analysis revealed homozygous deletions of Exon 2 & 4 of *CDKN2A* and Exon 2 of *CDKNA2B* gene. These genes are involved in cell cycle regulation and homozygous deletion carries poor prognosis in ALL.^16^

There are two reported cases of double-hit lymphoma expressing Tdt and presenting as ascites.^17^^,^^18^ Unlike ours, one case was a transformation from follicular lymphoma, and the other had involvement of the pancreas primarily. Primary effusion lymphoma (PEL) driven by HHV-8 is another differential. PEL cells typically display a “null” lymphocyte phenotype, i.e., CD45 is expressed, but routine B-cell (including surface and cytoplasmic immunoglobulin, CD19, CD20, CD79a) and T-cell (CD3, CD4, CD8) markers are absent. Instead, various markers of lymphocyte activation (CD30, CD38, CD71, human leukocyte antigen DR) and plasma cell differentiation (CD138) are usually displayed.^19^ Peritoneal lymphomatosis is treated non-surgically and often shows dramatic improvement with chemotherapy. Therefore, early and precise diagnosis is of utmost importance. However, PL with double-hit phenotype has a belligerent course and a poor outcome despite appropriate treatment.^20^

Double-hit genetics (*MYC*, *BCL2* rearrangement) have inferior outcome with standard chemotherapy. This case highlights that double hit genetics can be seen in B-precursor lymphoma/leukemia and a separate classification of this entity may be beneficial for prognostication and may facilitate further research and design the novel therapeutic approaches such as MYC and BCL2 inhibitors.

## CONCLUSIONS

- There should be early institution of empirical treatment while awaiting diagnostic results as it has an aggressive course and poor outcome;
- De novo double-hit B-cell precursor leukemia/lymphoma needs separate recognition for facilitating further research and targeted therapy.
